# Comparison of PCR ribotyping and multilocus variable-number tandem-repeat analysis (MLVA) for improved detection of *Clostridium difficile*

**DOI:** 10.1186/1471-2180-11-217

**Published:** 2011-09-30

**Authors:** Hsiao L Wei, Chun Wei Kao, Sung H Wei, Jason TC Tzen, Chien S Chiou

**Affiliations:** 1 The Central Region Laboratory, Center for Research and Diagnostics, Centers for Disease Control, 5F 20 Wen-Sin South 3rd Road, Taichung City 40855, Taiwan; 2 Graduate Institute of Biotechnology, National Chung-Hsing University, 250 Guoguang Road, Taichung City 40277, Taiwan; 3 The Third Branch Office, Centers for Disease Control, Taiwan

## Abstract

**Background:**

Polymerase chain reaction (PCR) ribotyping is one of the globally accepted techniques for defining epidemic clones of *Clostridium difficile *and tracing virulence-related strains. However, the ambiguous data generated by this technique makes it difficult to compare data attained from different laboratories; therefore, a portable technique that could supersede or supplement PCR ribotyping should be developed. The current study attempted to use a new multilocus variable-number tandem-repeat analysis (MLVA) panel to detect PCR-ribotype groups. In addition, various MLVA panels using different numbers of variable-number tandem-repeat (VNTR) loci were evaluated for their power to discriminate *C. difficile *clinical isolates.

**Results:**

At first, 40 VNTR loci from the *C. difficile *genome were used to screen for the most suitable MLVA panel. MLVA and PCR ribotyping were implemented to identify 142 *C. difficile *isolates. Groupings of serial MLVA panels with different allelic diversity were compared with 47 PCR-ribotype groups. A MLVA panel using ten VNTR loci with limited allelic diversity (0.54-0.83), designated MLVA10, generated groups highly congruent (98%) with the PCR-ribotype groups. For comparison of discriminatory power, a MLVA panel using only four highly variable VNTR loci (allelic diversity: 0.94-0.96), designated MLVA4, was found to be the simplest MLVA panel that retained high discriminatory power. The MLVA10 and MLVA4 were combined and used to detect genetically closely related *C. difficile *strains.

**Conclusions:**

For the epidemiological investigations of *C. difficile*, we recommend that MLVA10 be used in coordination with the PCR-ribotype groups to detect epidemic clones, and that the MLVA4 could be used to detect outbreak strains. MLVA10 and MLVA4 could be combined in four multiplex PCR reactions to save time and obtain distinguishable data.

## Background

*Clostridium difficile *is the most commonly recognized cause of infectious nosocomial diarrhea [1]. Illnesses associated with *C. difficile *range from mild diarrhea to pseudomembranous colitis and toxic megacolon [2]. In the early 2000s, an emerging virulent strain, NAP1/027, caused hospital outbreaks in Canada [3], and later, strains of the same genotype were also found in the United States of America, Europe, and Asia [3-5]. To understand the spread of bacteria and identify clones with apparent increased virulence, several molecular methods for genotyping have been used to investigate *C. difficile *[6-10]. Multilocus sequence typing (MLST) is the "gold standard" for assessing population structure. Polymerase chain reaction (PCR) ribotyping has been used for the global analysis of related virulent strains based on a reference library involving 116 genotypes acquired since 1999, and has become the most common technique to represent the epidemic clone of *C. difficile *[11]. In addition, pulsed-field gel electrophoresis (PFGE), surface layer protein A gene-sequence typing (*slpA*ST), restriction endonuclease analysis (REA), and multilocus variable-number tandem-repeat analysis (MLVA) have been used for outbreak studies of *C. difficile *[7,8,12-14]. Among these techniques, MLVA panels exhibit a significantly higher discriminatory power (allelic diversity: 0.964) than PFGE, *slpA*ST, and PCR ribotyping [9]. As a result, MLVA has been the most commonly used to distinguish strains from different outbreaks, whereas PCR ribotyping and PFGE have mostly been used to detect long-term relationships among strains when compare to MLVA [15,16].

PCR ribotyping is performed using a PCR-based method to detect polymorphic sequences in the 16S-23S intergenic spacer region (ISR) in *C. difficile *[17]. The band-pattern data generated by this method is difficult to transport and to compare between laboratories [18,19]. Therefore, a few studies have tried to replace PCR ribotyping with other methods [19-22]. Typing of *slpA*, which is based on the S-layer gene sequence of *C. difficile*, recognizes only nine of the 14 PCR-ribotypes [22]. Recently, a highly discriminatory MLST method based on seven housekeeping genes (*adk*, *atp*A, *dxr*, *gly*A, *rec*A, *sod*A, and *tpi*) sequences was develop to allow genotyping of *C. difficile*; the resulting sequence type (ST) recognized 32 of 40 PCR-ribotypes [21]. To date, the tandem repeat sequences type (TRST) technique is the most concordant method; this method, which combines two variable tandem repeat sequences, resolved the phylogenic diversity at a level equivalent to PCR ribotyping [20]. The MLVA employs multiple variable-number tandem-repeat (VNTR) loci with varying levels of diversity to resolve genetic relationships. VNTRs with a high degree of diversity are used to differentiate closely related strains. In addition, recent research in *Staphylococcus aureus *and *Neisseria meningitidis *showed that VNTR loci with a lower degree of diversity can establish deeper phylogenetic relationships consistent with the MLST method, which is based on the slowly-mutating housekeeping gene sequences [23,24]. In the past, for *C. difficile*, the MLVA panel has been found a more discriminatory method than PCR-ribotyping [13,14]. In this study, we hypothesize that an MLVA panel with a lower combined allelic diversity may be more congruent to PCR ribotyping.

The purpose here was to determine a MLVA panel that could yield results in accordance with PCR ribotyping results. Serial MLVA panels were compared with PCR-ribotype groups based on an investigation of 142 *C. difficile *isolates. By combining more conserved VNTR loci, we found MLVA10 had excellent congruence with the epidemic clone. Moreover, a simple MLVA (MLVA4) with high discriminatory power was also proposed as a useful alternative. Therefore, MLVA10 and MLVA4 can be combined in four multiplex PCR reactions to save operation time when typing a large collection of isolates.

## Results

### Identification and characterization of VNTR loci in *C. difficile*

A total of 47 VNTR loci candidates were identified for *C. difficile*, and 40 were used for subsequent MLVA analysis (Table 1, Additional file 1). Initially, we found 1,526 tandem-repeat loci within *C. difficile *630 using the VNTRDB software [25]. After exclusion of repeatedly detected loci, tandem-repeat loci with a copy number size >2 bp and an amplicon size of <700 bp were analyzed for variability. Finally, 47 loci exhibiting variable alleles were identified. The allelic diversity, allele number, and typing ability of all 47 VNTRs from the 142 strains were determined. Several VNTR loci with additional or imperfect repeats were observed (Additional file 1). CDR59 amplicon exhibited two adjacent VNTR loci, while CDR60, cd5, cd6, cd7, and cd25 exhibited incomplete tandem repeats. To analyze these loci in the MLVA panels, alleles of these loci were represented by repeat array size instead of copy number, and the MLVA types were analyzed with minimum spanning tree (MST) using a categorical coefficient. VNTR loci with low typing ability and/or deletions were excluded, with the CDR5, cd8, cd28, and cd20 loci amplifying at only 70%, 77%, 79%, and 79%, respectively. Additionally, deletions in amplicons from cd16, cd19, and cd39 were found. Consequently, only 40 VNTR loci were used in the following experiments.

**Table 1:** Characteristics of 47 *C. difficile *VNTR loci

Locus	Repeat (bp)	*C. difficile *630^a^		P142		
		
		Location	Copy number	No. alleles	Simpson's allelic diversity	Typeability (%)
C6cd^b^	6	3239736-855	16	32	0.96	98
CDR4^b^	6	755721-942	37	38	0.96	97
CDR49^b^	7	3688632-750	17	22	0.94	99
CDR60^b, c^	17	677132-413	265	20	0.94	92
CDR9^b^	8	664660-747	6	20	0.93	83
CDR5^b^	8	692929-3017	11	13	0.9	70
CDR48^b^	7	167124-172	7	10	0.84	99
cd7^c^	7	941339-465	128	10	0.83	97
cd5^c^	17-19	828221-372	150	15	0.8	96
cd6^c^	42	917090-173	84	10	0.78	99
CDR59^b,c^	11	771338-403	167	11	0.76	99
cd25^c^	12	3748418-65	57	6	0.71	98
F3cd ^b^	3	1954915-935	7	5	0.7	100
H9cd ^b^	3	4116072-110	13	7	0.62	100
cd12	12	1578610-45	3	4	0.61	100
cd22	15	3035898-942	2	5	0.58	99
cd20	17	2913124-157	2	3	0.56	79
cd19	18	2724077-166	5	4	0.56	100
cd27	15	1662349-63	2	5	0.55	100
cd31	17	4261467-517	3	3	0.54	100
cd10	6	1366501-24	4	2	0.5	100
cd16	11	2004175-85	1	2	0.5	98
cd41	18	857052-105	3	3	0.49	100
cd29	16	2025983-6014	2	2	0.49	100
cd8	8	1216864-79	2	5	0.42	77
cd23	21	3157267-350	4	5	0.41	100
cd17	8	2062186-201	2	2	0.35	100
cd30	15	3095446-75	2	2	0.33	100
cd15	5	1909382-6	2	3	0.32	100
cd14	19	1908272-309	2	2	0.3	100
cd39	5	1021318	0	9	0.28	100
cd4	15	667998-8057	3	3	0.27	100
cd21	6	2982766-787	8	3	0.27	100
cd2	14	463809-36	2	2	0.25	100
cd40	5	209313-27	3	3	0.22	100
cd9	3	1268365-77	4	2	0.22	100
cd42	4	1818181-92	3	4	0.21	99
cd28	8	1821467-82	2	4	0.2	79
cd18	4	2611912-27	4	4	0.16	100
cd33	24	1563736-83	2	2	0.12	100
cd13	23	1833582-673	4	4	0.11	100
cd36	3	4231072-84	2	3	0.11	100
cd24	10	3621903-22	2	2	0.09	100
cd32	6	339734-45	2	2	0.06	100
cd35	6	3925113-24	2	2	0.03	96
cd34	6	2033446-57	2	2	0.03	99
cd38	7	811821-34	2	2	0.03	100

^a ^Accesstion number in Genbank is AM180355.

^b ^Identified previously by Marsh et al. [13] and van den Berg et al. [14].

^c ^This locus contains incomplete repeat and is denoted by the size of array.

### Capillary gel electrophoresis-based PCR ribotyping

**Figure 1:**
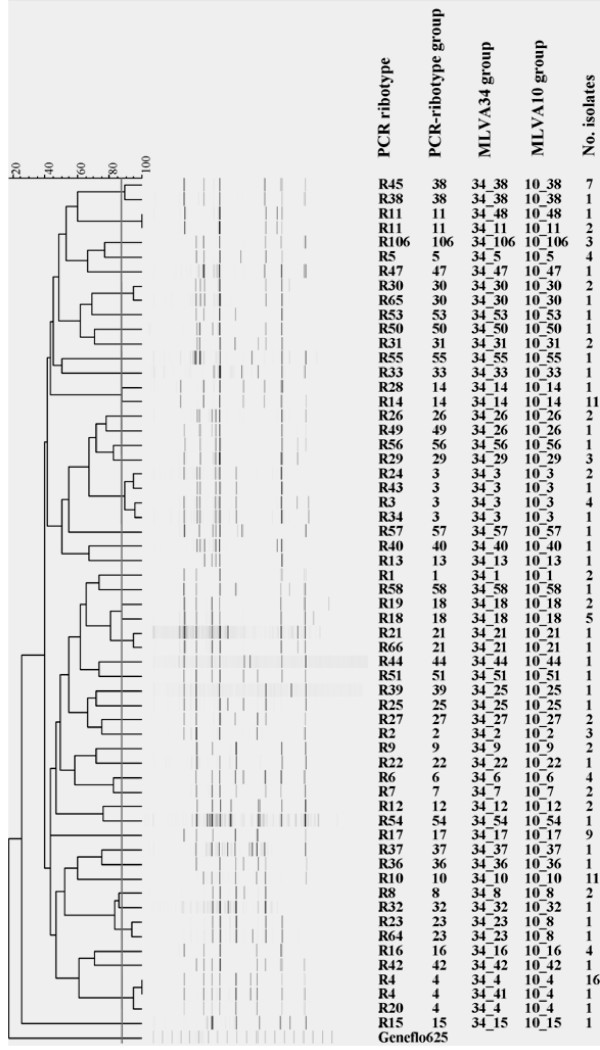
**Comparison of PCR riboytpe and MLVA groups for 142 C. difficile isolates**. Dendrogram is based on UPGMA analysis of capillary electrophoresis-based PCR ribotyping, and the vertical line is the cutoff point for identifying PCR-ribotype groups. Corresponding PCR-ribotype groups, MLVA34 groups, MLVA10 groups, and number of isolates are shown. MLVA groups are identified by minimum-spanning tree: one group is defined by MLVA type with less than two loci difference.

Of the 142 isolates, capillary gel electrophoresis-based PCR-ribotyping identified 57 independent types, including 32 singletons. The most common types were R45, R4, R10, R14, and R17 (UK017), containing 7, 17, 11, 11 and 9 isolates, respectively (Figure 1). The R27 (UK 027) virulent type was not found among the local strains.

### Dendrogram based on PCR ribotyping

A phylogenetic dendrogram based on the PCR-ribotypes was constructed using the 142 *C. difficile *isolates (Figure 1). Of the 142 isolates, PCR-ribotype, MLVA34, and MLVA10, identified 57 types, 47 groups, and 45 groups, respectively. The PCR-ribotype was more discriminatory than the two MLVA groups (Figure 1). Using a threshold of >83% similarity for defining PCR-ribotype groups, all isolates were able to be divided into 47 PCR-ribotype groups, including 22 singletons. Over 87% (41/47) of the PCR-ribotype groups were specifically recognized in the MLVA34 and MLVA10 groups. However, PCR-ribotype groups 39 and 25 were recognized together as one by both MLVA groups, with the fingerprints for these isolates sharing a 70% similarity (a four-band difference). In addition, PCR ribotype groups 26 and 49 were also identified as one by the two MLVA groups, with the fingerprints of these two isolates sharing a 78% similarity. Furthermore, PCR ribotype groups 8 and 23 were also seen as one by the two MLVA groups, with the fingerprint of these isolates sharing an 82% similarity. Taken together, these results shows that this discordance, the lack of one to one identification between PCR ribotypes and MLVA groups, mainly occurred when PCR-ribotypes shared >83% similarity.

### Congruence between groups of the PCR ribotype and MLVA

MLVA panels with slightly limit allelic diversity generated groups highly congruent with PCR ribotyping (Table 2). To determine the most congruent groupings between MLVA panels and PCR-ribotype groups, groupings of MLVA panels consisting of VNTR loci with high to low allelic diversity were compared with the PCR-ribotype groups. MLVA34, MLVA12, and MLVA10 generated partitions (47, 45, and 45, respectively) and allelic diversity (0.959, 0.957, and 0.957, respectively) similar to those identified by PCR ribotyping (Table 2). The congruence of the grouping of MLVA34, MLVA12, and MLVA10 with the PCR ribotype groups amounted to 97.3, 98.9, and 98.9%, respectively (Table 2). These values were significantly higher than that of the MLVA40 group (2.6%). MLVA40, which included six highly variable VNTR loci, C6cd, CDR60, CDR4, CDR49, CDR9, and CDR48 (allelic diversity: 0.84-0.96), generated a lot more partitions (136) and higher allelic diversity (0.999) than PCR ribotyping. In most PCR-ribotypes, multiple alleles were observed for C6cd, CDR60, CDR4, CDR49, CDR9, and CDR48 loci (Additional file 2), whereas the other 34 VNTR loci exhibited little variance. This data indicates that the greatest discrepancy between groupings in these two methods occurred in loci with high allelic diversity, and that congruence increased when the highly-allelic-diversity loci were removed, as in MLVA34.

**Table 2:** Congruence between PCR-ribotyping and MLVAs for grouping analysis of 142 *C. difficile *isolates

**Methods**^**a**^	No. partitions	**Simpson's ID **^**b**^	**Congruence **^**C**^	**95% CI**^**d**^
Ribotyping	47	0.957	1.000	
MLVA40	136	0.999	0.026	(0.185-0.354)
MLVA39	131	0.998	0.081	(0.000-0.172)
MLVA38	114	0.994	0.229	(0.099-0.362)
MLVA37	88	0.979	0.631	(0.487-0.789)
MLVA36	64	0.965	0.892	(0.822-0.969)
MLVA35	53	0.96	0.958	(0.918-1.000)
MLVA34	47	0.959	0.973	(0.932-1.000)
MLVA12	45	0.957	0.989	(0.973-1.000)
MLVA10	45	0.957	0.989	(0.973-1.000)
MLVA8	41	0.949	0.902	(0.823-0.968)

^a ^MLVA40: C6cd, CDR4, CDR49, CDR9, CDR60, CDR48, cd7, cd5, cd6, CDR59, cd25, F3cd, H9cd, cd12, cd22, cd27, cd31, cd10, cd41, cd29, cd23, cd17, cd15, cd30, cd14, cd4, cd42, cd2, cd40, cd9, cd18, cd36, cd33, cd13, cd354, cd24, cd34, cd32, cd21, cd38.

MLVA39: CDR4, CDR49, CDR9, CDR60, CDR48, cd7, cd5, cd6, CDR59, cd25, F3cd, H9cd, cd12, cd22, cd27, cd31, cd10, cd41, cd29, cd23, cd17, cd15, cd30, cd14, cd4, cd42, cd2, cd40, cd9, cd18, cd36, cd33, cd13, cd354, cd24, cd34, cd32, cd21, cd38

MLVA38: CDR49, CDR9, CDR60, CDR48, cd7, cd5, cd6, CDR59, cd25, F3cd, H9cd, cd12, cd22, cd27, cd31, cd10, cd41, cd29, cd23, cd17, cd15, cd30, cd14, cd4, cd42, cd2, cd40, cd9, cd18, cd36, cd33, cd13, cd354, cd24, cd34, cd32, cd21, cd38

MLVA37: CDR9, CDR60, CDR48, cd7, cd5, cd6, CDR59, cd25, F3cd, H9cd, cd12, cd22, cd27, cd31, cd10, cd41, cd29, cd23, cd17, cd15, cd30, cd14, cd4, cd42, cd2, cd40, cd9, cd18, cd36, cd33, cd13, cd354, cd24, cd34, cd32, cd21, cd38

MLVA36: CDR60, CDR48, cd7, cd5, cd6, CDR59, cd25, F3cd, H9cd, cd12, cd22, cd27, cd31, cd10, cd41, cd29, cd23, cd17, cd15, cd30, cd14, cd4, cd42, cd2, cd40, cd9, cd18, cd36, cd33, cd13, cd354, cd24, cd34, cd32, cd21, cd38

MLVA35: CDR48, cd7, cd5, cd6, CDR59, cd25, F3cd, H9cd, cd12, cd22, cd27, cd31, cd10, cd41, cd29, cd23, cd17, cd15, cd30, cd14, cd4, cd42, cd2, cd40, cd9, cd18, cd36, cd33, cd13, cd354, cd24, cd34, cd32, cd21, cd38

MLVA34: cd5, cd6, cd7, cd12, cd22, cd25, cd27, cd31, F3cd, H9cd, CDR59, cd10, cd41, cd29, cd23, cd17, cd15, cd30, cd14, cd4, cd2, cd42, cd40, cd9, cd18, cd36, cd33, cd13, cd35, cd24, cd34, cd32, cd38, cd21.

MLVA12: cd5, cd6, cd7, cd12, cd22, cd23, cd25, cd27, cd31, F3cd, H9cd, CDR59.

MLVA10: cd5, cd6, cd7, cd12, cd22, cd27, cd31, F3cd, H9cd, CDR59.

MLVA8: cd5, cd6, cd7, cd12, cd27, F3cd, H9cd, CDR59.

^b ^Simpson's allelic diversity.

^c ^Adjusted Rand's coefficient.

^d ^95% CI, 95% confidence interval of ongruence.

To identify a simplified panel resembling MLVA34, the groups from three smaller panels (MLVA12, MLVA10, and MLVA8) were evaluated for agreement with the PCR-ribotype groups. MLVA10 was the simplest panel yielding groups that were highly congruent (98%) with the PCR-ribotype groups (Table 2). In contrast, congruence significantly decreased when the MLVA was simplified to just eight VNTR loci.

### Minimum spanning tree analysis of PCR ribotyping-related MLVA panels

**Figure 2:**
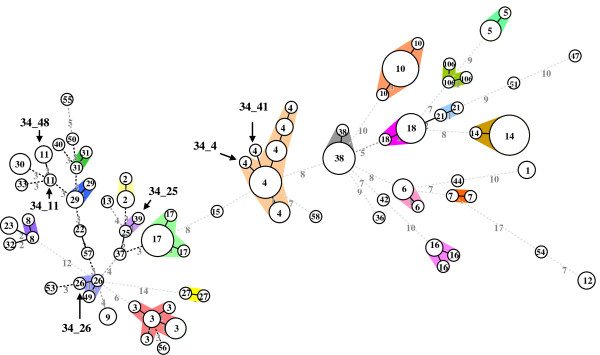
**Minimum-spanning tree of MLVA34 data from 142 *C. difficile *isolates**. Each circle represents unique MLVA type. The numbers between circles represent the VNTR loci differences between MLVA types. The numbers inside circles represent the PCR-ribotype groups. MLVA groups were defined as MLVA types having a maximum distance changes at one loci. The different shaded colors denote isolates belonging to a particular MLVA groups. Hyphenated numbers represent the MLVA groups marked with arrows.

MST analysis revealed that the MLVA34 types could be clustered into 47 groups, including 21 singletons (Figure 2). Most (41/47) of the MLVA34 groups were specifically recognized as a single PCR-ribotype group, except for 34_4, 34_41, 34_11, 34_48, 34_25, and 34_26. An isolate of the group 34_41 could not be typed by the cd7 and cd34 loci, and was separated from those of the 34_4 MLVA group; however, all isolates of the 34_41 and 34_4 groups belonged to PCR-ribotype group 4. This shows that isolates of the 34_4 and 34_41 groups were closely related. Isolates of group 34_11 and 34_48 were separated by their different allele numbers at CDR59 and H9cd loci, but these two MLVA groups both belonged to the PCR-ribotype group 11.

**Figure 3:**
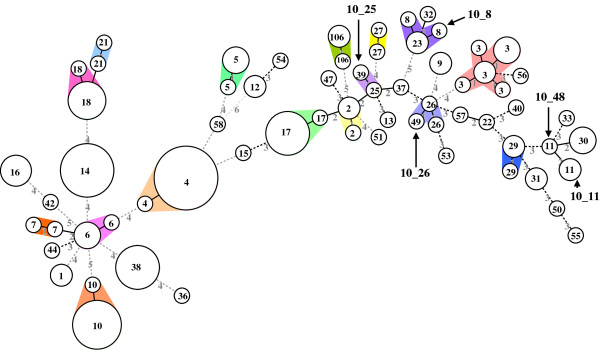
**Minimum-spanning tree of MLVA10 data from 142 *C. difficile *isolates**. Each circle represents unique MLVA type. The numbers between circles represent the VNTR loci differences between MLVA types. The numbers inside circles represent the PCR-ribotype groups. MLVA groups were defined as MLVA types having a maximum distance changes at one loci. The different shaded colors denote isolates belonging to a particular MLVA groups. Hyphenated numbers represent the MLVA groups marked with arrows.

MST analysis revealed that the MLVA10 types could be clustered into 45 groups, including 20 singletons (Figure 3), and most (41/45) of the MLVA10 groups were specifically recognized as a single PCR-ribotype group. The clustering of MLVA10 (Figure 3) yielded groupings similar to those of MLVA34, except for isolates of PCR-ribotype groups 4, 8, and 23. Since the cd34 VNTR locus was not used in the MLVA10 panel, isolates from the PCR-ribotype group 4 all belonged to the 10_4 group. This indicates that the MLVA10 panel was able to type more strains than the MLVA34 panel. In addition, isolates of the PCR-ribotype groups 8 and 23 were grouped into the 10_8 group, indicating that the MLVA10 is less discriminatory than MLVA34.

### Discriminatory ability of MLVA panels

MLVA panels containing different numbers of VNTR loci were used for discriminating 142 *C. difficile *isolates into different genotypes and the Simpson's index of diversity (ID) was shown to increase with the number of VNTR loci used (up to MLVA4; Table 3). Using MLVA4, 142 isolates were grouped into the largest partitions (140). MLVA4 was shown to be as discriminatory as MLVA40 using all 40 VNTR loci (Table 3). However, when the MLVA panels contained fewer than three VNTR loci, the partitions decreased significantly.

**Table 3:** Comparison of discriminatory power for PCR-ribotyping and MLVAs based on various combinations of VNTR loci

**Method**^**a**^	No. genotypes	**Simpson's ID **^**b**^	**95% CI **^**c**^
Ribotype	57	0.9640	0.9515-0.9766
MLVA2	126	0.9983	0.9972-0.9994
MLVA3	139	0.9997	0.9992-1.0002
MLVA4	140	0.9998	0.9994-1.0002
MLVA6	140	0.9998	0.9994-1.0002
MLVA40	140	0.9998	0.9994-1.0002

^a ^MLVA2: C6cd, CDR4.

MLVA3: C6cd, CDR4, CDR49.

MLVA4: C6cd, CDR4, CDR49, CDR60.

MLVA6: C6cd, CDR4, CDR49, CDR60, CDR9, CDR48.

MLVA40: C6cd, CDR4, CDR49, CDR60, CDR9, CDR48, cd7, cd5, cd6, cd25, CDR59, F3cd, H9cd, cd12, cd22, cd27, cd31, cd10, cd41, cd29, cd23, cd17, cd15, cd30, cd14, cd4, cd42, cd2, cd40, cd9, cd18, cd36, cd33, cd13, cd35, cd24, cd34, cd32, cd21, cd38.

^b ^Simpson's allelic diversity.

^c ^95% CI, 95% confidence interval of Simpson's ID.

### Combined use of MLVA4 and MLVA10 for cluster detection

**Figure 4:**
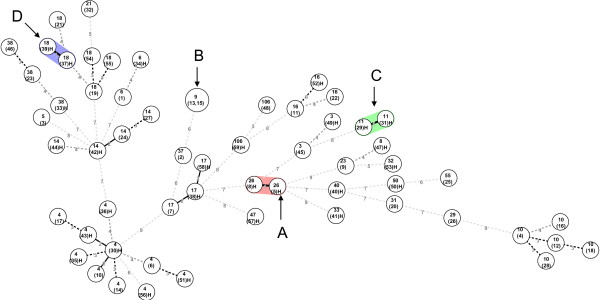
**Minimum-spanning tree of MLVA10 and MLVA4 data from 60 *C. difficile *isolates from inpatients**. Each circle represents unique MLVA type. The numbers between circles represent the VNTR loci differences between MLVA types. The numbers inside circles represent the PCR-ribotype groups. The numbers in parentheses inside circles denotes the strain number. MLVA types isolated from inpatient are labeled with an "H". One cluster was defined as MLVA types having a maximum distance changes at one loci. The different shaded colors denote isolates belonging to a particular cluster. Clusters marked with arrows are labeled by alphabetical order.

MLVA4 and MLVA10 were used for classifying 59 isolates acquired from a hospital in central Taiwan, and four clusters were identified (Figure 4; Additional file 3). These clusters consisted of three independent clusters (B, C, and D) containing two isolates each from inpatients and one (A) cluster containing two isolates from outpatients during the ten month surveillance. Each of the two isolates from the B, C, and D clusters were recovered from different pediatric patients with 3, 0, and 4-days intervals of specimen submission by the physician from children's ward, respectively (Additional file 3). The two isolates from cluster A were shown to differ at one locus (1/14) in the combined MLVA4 plus MLVA10 panel and were isolated from two specimens of the same patient within a four-day interval. Most isolates were non-toxigenic strains, except those in cluster D. The patient in the D cluster developed diarrhea and was infected with toxigenic *C. difficile *strains that were assigned to *C. difficile *infection cases. On the other hand, a single PCR-ribotype group was usually grouped with less than five VNTR loci differences (5/14).

## Discussion

A MLVA system is composed of VNTR loci that exhibit varying levels of diversity, and can be employed either for long-term or short-term investigations [26]. In the present study, we proposed two MLVA panels, MLVA10 and MLVA4, for the differentiation of *C. difficile *isolates. MLVA10 exhibited a slightly lower allelic diversity than previously identified panels [13,14], and is recommended as a complementary test to the PCR-ribotype groups. MLVA4, in contrast, exhibited high allelic diversity and is recommended for the detection of short-term evolution in strains of *C. difficile*.

In the current study, except for nine reference strains, the 133 local isolates were a widely distributed collection and none were previously reported as outbreak strains by clinical laboratories. These isolates were acquired from patients 0.1-88 years of age and contained 73 isolates from outpatients that were assumed to be community-acquired strains. The other 60 isolates were recovered from hospitalized patients, with 38 collected from children's wards and 22 from adult wards. In addition, this study involved 57 PCR-ribotypes (Table 3), a considerably higher number than previously reported [9]. Therefore, the sample population used in the current study is proposed to be more suitable for comparison between the two methods [20,21,27]. In the ribotype distribution, it is noteworthy that the PCR-ribotype R17 (UK 017), a clone found worldwide and is related to an animal source (in addition to 027 and 078 types) was the fourth (9 in 142) most frequently identified type in this study (Figure 1) [28,29]. In the current study, the R17 type was only found in samples obtained from central Taiwan, but the exact distribution of PCR-ribotypes requires further investigation using a more precise sampling method. Furthermore, PCR-ribotypes other than 001, 017, 027, and 106 should be compared with standard PCR-ribotypes from the European reference laboratory.

While comparing PCR ribotyping to other techniques, allelic diversity was identified as an important factor. Previous studies identified that *slpA *type did not have high enough variability to differentiate all PCR-ribotypes [22]. The current study found that the CDR4, CDR9, CDR48, CDR49, CDR60, and C6cd VNTR loci [13,14,19] used in previous MLVA panels were variable in each PCR-ribotypes (Additional file 2); this made these panels too discriminatory for congruency with the PCR-ribotypes here. In contrast, the highly discriminatory MLST method had an index of discrimination of 0.9, similar to that of the PCR-ribotype (0.92), and the resulting ST recognized 80% of the PCR-ribotypes [21]; the TRST resulted in an allelic diversity (0.967) equal to that of PCR ribotyping (0.967), and is the technique most related to PCR ribotyping among these studies [20]. In the present study, the ten VNTR loci used in MLVA10 were cd5, cd6, cd7, cd12, cd22, cd27, cd31, H9cd, F3cd, and CDR59, which exhibited a slightly lower allelic diversity (0.54-0.83) than the previously used CDR4, CDR9, CDR48, CDR49, CDR60, and C6cd VNTR loci (0.84-0.96) [13,14,19,20] (Table 1), resulting in a combined allelic diversity of 0.957 (Table 2). This value is similar to TRST (0.967) and PCR-ribotype (0.967). Therefore, both TRST and MLVA10 showed a high level of agreement with the PCR-ribotype (86.0 and 88.2%, respectively) (Table 2). However, the MLVA technique is easier to perform than the sequence-based techniques, such as TRST and MLST, and MLVA panels are more easily combined, such as when adding the MLVA4 panel for outbreak strain detection.

To represent the currently known PCR-ribotypes for *C. difficile*, a combination of multiple VNTR loci with different allelic diversity is recommended. In our initial study, no single VNTR locus was discriminatory enough to recognize all PCR-ribotypes or specific enough to belong to each PCR-ribotype (data not shown), as previously observed for MLVA and MLST of *N. meningitidis *[24]. Therefore, 40 VNTR loci distributed throughout the genome of the *C. difficile *630 strain were used for comparison analyses, and we found that the MLVA34 panel yielded groups most related to the PCR-ribotype groups (Table 2; Figure 1). Our screening method was based on two rationales: 1) the PCR-ribotype recognized the major PFGE type [9] and was expected to be congruent with the major genotypic groups of *C. difficile*; and 2) the locus markers distributed throughout the chromosome were more likely to identify genotypic change [13].

In the current study we also highlighted the fact that group definition was required for comparisons. The allelic diversity of MLVA10 types varied among the different PCR-ribotypes (Additional file 4), and led to only 60% congruence between the types of MLVA10 and PCR ribotyping (data not shown). In significant contrast, the congruence reached 98% when groups obtained by the two techniques were compared (Table 2). These observations were similar to those found in the comparison between MLVA34 and PCR-ribotyping (Additional file 4). Even though there was a high level of agreement between groups identified by the two techniques, some discordance was found. For example, PCR-ribotype group 11 was represented by two MLVA10 groups (10_48 and 10_11) (Figure 1), and the isolates in group 11 were suspected to have undergone concerted evolution [30,31]; however, this assumption needs to be further confirmed by MLST.

For the detection of outbreak strains, two MLVA panels, each composed of seven VNTR loci, have been developed. One panel consisted of CDR4, CDR5, CDR9, CDR48, CDR49, CDR59, and CDR60, and the other panel consisted of C6cd, H9cd, F3cd, CDR4, CDR9, CDR48, and CDR49 [13,14]. However, our study indicated that MLVA4, which consisted of C6cd, CDR4, CDR49, and CDR60, was able to discriminate all 142 test strains (Table 3), as previously observed for MLVA of *Salmonella typhimurium *[32]. Furthermore, all of these VNTR loci exhibited higher allelic number and copy number variation than previously reported (Table 1) [14]. Our results may be explained by two reasons: 1) among these loci, CDR60 loci was found exhibit incomplete copy number and was assigned by repeat array size, as this could increase the allelic number; and 2) we validated these loci in a more random population than previous studies [13,14], which would increase the value of allelic diversity. In addition, we used a categorical coefficient instead of STRD to analyze the MLVA data and to analyze the loci represented by the repeat array size. Although this may reduce the sensitivity to differentiate the outbreak strains, analyses using the STRD coefficient were found to be too variable and may obscure the epidemiological links between *C. difficile *outbreak strains when several repeats at a locus are deleted or duplicated simultaneously [33].

All clusters detected by MLVA4 and MLVA10 combined can be explained by epidemiological information. Apart from the two patients from cluster D were *C. difficile *infection cases, other patients from other clusters were assumed to be *C. difficile *carriers (Figure 4; Additional file 3). The major limitation of this validation for the study of outbreak strains was the sample population we used; the 142 test strains used in the current study were a randomly sampled population that did not contain outbreak strains, and the genetic relationship between these was distant. For these reasons, this may have overestimated the discriminatory power of the MLVA 4. Therefore, the MLVA4 panel requires further validation using closely related strains, such as outbreak strains from hospitals, before any conclusions as to its discriminatory power can be made.

Five imperfect VNTR loci (cd5, cd6, cd7, CDR59, and CDR60) were used in this study, except for CDR59, the other four loci were long-repeat VNTR loci with incomplete repeats (Additional file 1). The incomplete repeats may be caused by insertions and deletions, which often result in horizontal gene transfer between bacteria strains and obscured the phylogenic relationship in the bacteria population [34]. However, the long-repeat regions exhibited a higher frequency of recombinations, and were considered attractive candidate regions that could be used for determining phylogenetic relatedness between species and strains [35]. The long-repeat VNTR loci have been known to be responsible for adaptive evolution, as for antigenic variation [34], and were also used to differentiate the *C. botulinum *and *N. meningitides*[36,37]. Therefore, we analyzed these imperfect VNTR loci for use in the screening for appropriate panels that showed agreement PCR-ribotyping. Our data showed that cd5, cd6, and cd7 loci did not decrease the congruency with PCR-ribotyping (Table 2; Additional File 2). The result may be due to that the 16S-23S intergenic spacer region, on which the PCR-ribotyping based on, was not as conserved as a housekeeping gene that is used to construct the phylogenic tree [9,38]. However, the variations from these incomplete repeat loci should be detected in our follow-up surveillance.

PCR ribotyping is a standard technique used worldwide for epidemic clone detection, but the ambiguous data generated by this technique is difficult for assessing inter-laboratory efficacy. MLVA is a fast and easy-to-use method, and its numerical profile output is more transferable than the standard PCR ribotyping technique. In our laboratory setting, the cost of PCR ribotyping, MLVA10, and TRST per isolate was $0.87, $2.53, and $13.60, respectively, and the cost of the most recent MLST is $24.65 according to Griffiths' estimation [21]. In the current study, the cost of MLVA10 was slightly higher than that of PCR ribotyping, but was still significantly less expensive than the TRST and MLST sequence-based typing techniques. Moreover, when analyzing a large number of isolates, it is simpler to perform one genotyping technique than multiple techniques. Taken together, the MLVA10 is recommended for the detection of *C. difficile *PCR-ribotype groups and for use in combination with the MLVA panel designed for the detection of outbreak strains. Future studies will involve evaluation of MLVA10 for its phylogenetic information by comparison to MLST typing.

## Conclusions

For the classification of *C. difficile *strains, the MLVA technique can result in a distinguishable data set that is more useful for comparison and is highly congruent with PCR-ribotype results. The MLVA10 panel may be used either to detect the PCR-ribotype groups or to overcome the drawbacks of the PCR ribotyping technique. In addition, the MLVA4 can be used to detect closely-related strains. These two MLVA panels can be combined and used for epidemiological studies of *C. difficile*.

## Methods

### Bacterial strains

A total of 142 *C. difficile *strains that were either toxigenic or non-toxigenic were used in this study. Five reference strains (NCTC11204, NCTC13366, NCTC13287, NCTC13404, and NCTC13307) were purchased from the National Collection of Type Cultures (NCTC, London, UK) and three reference strains (BCRC17900, BCRC17702, and BCRC17678) were purchased from the Bioresource Collection and Research Center (BCRC, Hsinchu, Taiwan). One strain (NAP1/027) was kindly provided by Dr. Brandi Limbago from the United States Centers for Disease Control and Prevention (CDC), and 133 strains were isolated from clinical laboratory specimens in Taiwan. Among local isolates, 73 strains were isolated from outpatients, and 60 strains were isolated from hospitalized patients that were comprised of 38 from adult wards and 22 from children's wards.

### Specimen, epidemiological data collection, and bacterial isolation

All specimen strains were provided by five clinical laboratories between November 27, 2007 and December 31, 2008. The corresponding epidemiological data for each strain were provided by clinical laboratory staff. Four laboratories were located in central Taiwan, and one laboratory in the southern part of Taiwan. All five clinical laboratories cultured all available stool or rectal-swab specimens on Cycloserine Cefoxitin Fructose Agar (Oxoid, Hampshire, UK) and the plates were incubated under anaerobic conditions for 48 h. All suspected *C. difficile *colonies were sent in an anaerobic pack and delivered within 24 h to the central-region laboratory at the Centers for Disease Control in Taiwan for further identification. All purified isolates were stored in 15% glycerol at -80°C.

### Isolate identification and toxigenic-type characterization Text for this sub-section

All suspected *C. difficile *colonies were analyzed for a species-specific internal fragment of the triose phosphate isomerase (*tpi*) housekeeping gene, and toxigenic type was characterized by PCR amplification of internal fragments of the toxin A gene (*tcdA*) and the toxin B (*tcdB*) gene, as previously described [39]. Briefly, each candidate colony was dissolved in 1 mL distilled water and then boiled for 15 min to prepare DNA. *Tpi-*, *tcdA*-, and *tcdB*-specific primers [39] were used in independent PCR reactions. PCR was performed in 20 μL volumes containing the following components: 50 ng DNA, 10% glycerol, 0.5 μM of each primer, 200 μM dNTPs, and 1 U of Taq DNA polymerase (BioVan, Taiwan) in a 1× amplification buffer solution (10 mM Tris-HCl [pH 8.3], 50 mM KCl, and 1.5 mM MgCl_2_). PCR was performed on a GeneAmp System 2400 thermal cycler (Applied Biosystems). The PCR cycle conditions were as follows: 95°C for 3 min, followed by 30 cycles of 95°C for 30 s, 55°C for 30 s, and 72°C for 30 s, and a final extension at 72°C for 3 min. PCR products were resolved by electrophoresis on a 1.5% agarose gel stained with ethidium bromide.

### VNTR identification and selection

The full-length sequences of *C. difficile *QCD-32g58 and *C. difficile *630 were compared using VNTRDB software [25] to find tandem repeat loci in the genome. Tandem repeats with a repeat length >2 bp and ≥70% consensus match were initially selected for screening by PCR from the BCRC17678 and BCRC17702 reference strains and four experimental isolates. Primers that flanked the tandem repeat region were designed using the online Primer 3 software (http://frodo.wi.mit.edu/primer3; Additional file 5). VNTR screening was initially performed by PCR amplification of each candidate tandem repeat locus in genomic DNA from six isolates. The variability of each tandem repeat locus was assessed by gel electrophoresis on a 1.5% agarose gel, and sequence analysis was performed to determine the size of the resulting PCR products and the tandem repeat copy number.

To find a MLVA panel most congruent to PCR ribotyping, 40 VNTR loci were sorted by allelic diversity and then arranged to form various panels by sequentially removing the highest allelic diversity loci. Each panel was compared with PCR ribotyping, and the congruence between the two techniques was calculated using the Rand coefficient [40].

The simplest MLVA panel that would yield a MLVA34-like genotype distribution of 142 *C. difficile *strains was found as follows. First, the partitions given by each of the 34 VNTR loci were calculated based on Wallace coefficients to evaluate their predictable value by the other 33 loci. Loci that showed either more predictability or lower allelic diversity than other loci in the MLVA34 panel were excluded. There were 22, 24, and 26 loci excluded when the predictable values were higher than 75, 70, and 65%, respectively. This exclusion resulted in the MLVA12, MLVA10, and MLVA8 panels (Additional file 6). All MLVA panels were analyzed by the minimum spanning tree (MST) method, and the concordance between MLVA groupings and PCR-ribotype data were calculated.

### DNA preparation

Genomic *C. difficile *DNA was purified using the QIAamp DNA Mini kit (QIAGEN, Hilden, Germany), according to the manufacturer's instructions. Genomic DNA isolated from *C. difficile *were then used for PCR amplification of VNTR and PCR ribotyping.

### Sequence analysis

PCR amplification of the 47 VNTR candidates was performed on six strains with the primer sets shown in Table 1. Each PCR was performed in a 10 μL reaction containing the following reagents: 25 ng genomic DNA, 1 μL buffer (10 mM Tris-HCl [pH 8.3], 50 mM KCl, and 1.5 mM MgCl_2_; BioVan, Taiwan), 250 μM MgCl_2_, 1% DMSO (Sigma-Aldrich, St. Louis, MO), 200 μM dNTPs, 0.5 μM primer set, and 1 U Taq DNA polymerase (BioVan, Taiwan). The PCR cycle conditions were as follows: 94°C for 5 min, followed by 30 cycles of 94°C for 40 s, 50°C or 52°C for 90 s, and 72°C for 50 s, and a final extension at 72°C for 3 min. Sequence analysis of the PCR products was performed by Mission Biotech Corporation with the ABI Big Dye Terminator Kit v.3.1 (Applied Biosystems) and the same primers used for PCR.

### Multilocus VNTR amplification

PCR amplification of the 48 selected *C. difficile *VNTR loci was performed on DNA extracted from 142 *C. difficile *isolates. The primer sets, annealing temperatures, and primer panels are shown in Additional file 5. Amplification of the 47 VNTR loci was carried out in 12 multiplex PCR reactions and one single PCR reaction (Additional file 5: M1-M13). Amplification of the 14 VNTR loci of MLVA4 and MLVA10 was carried out in four multiplex PCR reactions (Additional file 5: M14-M17). The PCRs were performed in 10 μL reactions containing the following reagents: 25 ng genomic DNA, 1 μL buffer (10 mM Tris-HCl [pH 8.3], 50 mM KCl, and 1.5 mM MgCl_2_; BioVan, Taiwan), 250 μM MgCl_2_, 1%DMSO (Sigma-Aldrich, St. Louis, MO), 200 μM dNTPs, 0.02-0.1 μM primer set, and 1 U Taq DNA polymerase (BioVan, Taiwan). The PCR cycle conditions were as follows: 94°C for 5 min, followed by 30 cycles of 94°C for 40 s, annealing temperature for 90 s, and 72°C for 50 s, and a final extension at 72°C for 3 min. Fragment analysis of the multiplex PCR products was performed as follows: 1 μL of each 20-fold-diluted PCR product, 0.1 μL GeneScan 500 LIZ size standard (Applied Biosystems, Warrington, UK) and 8.9 μL HiDi (Applied Biosystems, Foster, CA) were mixed and denatured at 95°C for 5 min. The products were then analyzed on an ABI3130 sequence detection system (Applied Biosystems). The obtained fragment sizes were exported as an Excel spreadsheet file (Microsoft, Redmond, WA). The corresponding copy numbers were calculated by comparison to the size of reference strains using Excel software (Microsoft). The equation used for calculation of copy number is as follows:

Copy number of VNTRn = [(Fs-Fr)/repeat size of VNTRn] + copy number of reference strain, where Fs, fragment size of test strains in each VNTR loci; Fr, fragment size of reference in each VNTR loci; VNTRn, either locus in 40 VNTR loci.

### Capillary gel electrophoresis-based PCR ribotyping

Genomic DNA from all the *C. difficile *strains was amplified with the primer set designed by Bidet *et al*. [18], and the electrophoresis-based PCR-ribotyping was performed using a method modified from Indra *et al*. [19]. Briefly, the primer was labeled with carboxyfluorescein (FAM) dye to enable DNA sequence analysis. The PCR mixture included the following reagents: 25 ng genomic DNA, 1 μL buffer (10 mM Tris-HCl [pH 8.3], 50 mM KCl, and 1.5 mM MgCl_2_; BioVan, Taiwan), 200 μM dNTPs, 1.5 mM MgCl_2_, and 1 U Taq polymerase (BioVan, Taiwan) in a 20 μL final volume. One microliter of each 20-fold-diluted PCR product, 0.8 μL Genflo625 ROX-labelled DNA Ladder (Chimerx, USA), and 8.2 μL HiDi (Applied Biosystems, Foster, CA) were mixed and denatured at 95°C for 5 min and then analyzed with a ABI3130 sequence detection system. The ribotype fragments for the full-length sequencing of strain NCTC13307 (*C. difficile *630) were first predicted by the PCR-amplification function from *in silico *analysis using the website (http://insilico.ehu.es), and the curve file from the ABI sequencer was confirmed by the predicted size. Ribotypes 001, 012, 017, 027, and 106 were set up by comparing the curve files with the five reference strains NCTC11204, NCTC13307, NCTC13366, NCTC 13287, and NCTC13404, respectively. All PCR-ribotypes were named with an "R" prefix before the serial number.

### Allelic diversity and typeability measurement

The allelic diversity of each VNTR locus was measured by its Simpson's index [41] and confidence interval (CI) [42]. The ability of each VNTR locus to type the 142 isolates was measured as follows:

Number of isolates amplified in each VNTR locus/142.

### Data analysis

The copy numbers of the VNTR loci from all of the 142 isolates were imported into the Bionumerics software (Applied Maths, Belgium), and the categorical coefficient and the highest number of single-locus-changes were used for the minimum spanning tree construction [43]. The curve files of all the ribotypes from the ABI sequencer were imported into the Bionumerics software for further standardization. The PCR-ribotyping fingerprints of all the isolates were analyzed using the Unweighted Pair Group Method with Arithmetic Mean (UPGMA) clustering algorithm, using the Dice coefficient (tolerance: 0.2%). The quantitative level of congruence between the typing techniques was based on the adjusted Rand (AR); the predictable value between VNTR loci was based on Wallace's coefficients, using an online tool for the quantitative assessment of classification agreement (http://darwin.phyloviz.net/ComparingPartitions) [40].

## Authors' contributions

HLW and CWK performed the microbiological and molecular studies. HLW and JT analyzed the data. HLW and CSC designed the research and wrote the manuscript. SHW collected and analyzed the epidemiological data. HLW and CWK revised the manuscript. All authors read and approved the final manuscript.

## Supplementary Material

Additional file 1 **Copy numbers, fragment sizes, sequences, and GenBank accession number of each allele at 40 VNTR loci**. This table provides the copy number and fragment sizes of the six initially test strains. The copy numbers (or array sizes) in each allele, their corresponding sequence, and their GenBank accession number are shown. Click here for file

Additional file 2 **Allelic number and allele of VNTR loci in each PCR ribotype**. This table provides the allelic number and allele of VNTR loci in each PCR ribotype, and only allelic number larger than one are listed. Click here for file

Additional file 3 **Epidemiological data, toxigenic type, and molecular type of isolates from one hospital in central Taiwan**. This table provides the molecular typing data of MLVA10 and MLVA4 for *C. difficile *isolates from one hospital in Taiwan, and the corresponding epidemiological data and characteristic of each strain are shown. Click here for file

Additional file 4 **Allelic diversity of MLVAs in each PCR ribotype**. This table provides the Simpson's allelic diversity of either types or groups from MLVA10 and MLVA34 panels. Click here for file

Additional file 5 **Primers for amplification of each locus**. This table provides a list of primers, annealing temperature, and primer concentration for amplification of each VNTR loci. Click here for file

Additional file 6 **List of predictable VNTR loci at 75%, 70%, and 65% predictable value**. This table provides the list of VNTR loci which could be predicted by loci in MLVA12, MLVA10, and MLVA8. Click here for file
